# Resprouting Response among Savanna Tree Species in Relation to Stem Size, Woody Removal Intensity and Herbicide Application

**DOI:** 10.3390/plants12193451

**Published:** 2023-09-30

**Authors:** Piet Monegi, Ntuthuko Raphael Mkhize, Julius Tlou Tjelele, David Ward, Zivanai Tsvuura

**Affiliations:** 1 Animal Production, Agricultural Research Council, Private Bag X 02, Irene 0062, South Africa; 2 School of Life Sciences, University of KwaZulu-Natal, Scottsville 3209, South Africa; 3 Animal and Poultry Science, School of Agriculture, Earth and Environmental Sciences, University of KwaZulu-Natal, Scottsville 3209, South Africa; 4 Department of Biological Sciences, Kent State University, Cunningham Hall, Kent, OH 44242, USA

**Keywords:** Picloram, savanna, stem diameter, tree cutting, woody plant encroachment

## Abstract

Mechanical and chemical methods are widely used to control woody plant encroachment in many African countries. However, very little is known about the effectiveness of these control methods among woody species of different ages. We conducted a field experiment to determine the effects of different tree removal treatments (10%, 20%, 50%, 75% and 100%) and herbicide application (Picloram; 6 mL L^−1^) on the resprouting ability and vigour of 12 woody plant species. We examined 20 plots (30 m × 30 m) that were each subjected to tree removal, followed by herbicide application on half of the stems for each plot. All the tree species in this study resprouted after cutting. The applied concentration of herbicide significantly reduced the shoot production for *Ehretia rigida*, *Vachellia robusta* and *Ziziphus mucronata*, with a marginal effect for *Dichrostachys cinerea*. The diameter of stems was an important factor in determining resprouting ability, with shoot production decreasing with increasing stem diameter. However, stem diameter did not affect shoot length and diameter for all species. We found that woody plants are more likely to resprout and survive as juveniles than as adults after cutting and that herbicide only affected four of the twelve species at a concentration of 6 mL L^−1^. Thus, testing the amount of Picloram needed to kill certain woody species may be of importance for land users in southern African savannas.

## 1. Introduction

Woody plant encroachment is considered one of the most extensive forms of rangeland degradation in arid and semi-arid areas globally [1,2] and can drastically reduce forage production for livestock and wild animals [3,4,5]. Woody plant proliferation is exacerbated in rangelands overgrazed by, among others, large herbivores, climate change and the suppression of fire that is used to control tree establishment in savanna ecosystems, as well as combinations of these factors [6]. In southern African savannas, the proliferation of woody plants is facilitated by leguminous trees such as the *Vachellia* and *Senegalia* species [7] and also by broadleafed species [8]. Woody plants have encroached on over 7.3 million hectares in South Africa [9]. This has led to a considerable reduction in plant diversity and grazing capacity, owing to reduced herbaceous species richness and biomass [10,11].

To properly manage and sustain the economic viability of savanna rangelands affected by woody plant encroachment, it is important to encourage the ecological benefits of woody plants in terms of the nitrogen fixation of leguminous trees [12], hydraulic lift [13,14] and organic carbon [15,16] while limiting their encroachment [17,18]. Effective rangeland management can be achieved by developing appropriate strategies that can help increase or maintain grass production adequate for livestock and game ranching [19,20]. One strategy for optimising the availability of grass and maintaining the ecological benefits conferred by woody plants is reducing tree density (also termed *tree thinning*), which involves a reduction in the number of trees in areas where woody plant encroachment has occurred [3]. Tree density reduction has been shown to have positive benefits in savannas, such as an increase in grass production and reducing soil erosion [3,21].

Globally, brush management techniques may include mechanical and chemical control methods to remove most of the woody layer [20,22,23,24]. A problem that is widely understood is that mechanical control methods are limited by the fact that many trees resprout after disturbances [25,26]. Many empirical studies have demonstrated the importance of resprouting as a persistence strategy across different habitats, from savannas [27,28,29], forests [30,31,32] and deserts [33] to Mediterranean ecosystems [31,32]. Resprouting is a mechanism that allows individual plants to regenerate after the elimination of the above-ground biomass and persist in ecosystems with recurrent disturbances [30,34]. Woody plants have been reported to regenerate from the cut or broken stem [34,35]. The resprouting ability of various woody plants is supported by the non-structural carbohydrate reserves stored in a well-developed, deep-root system [30,36]. Woody plants with strong resprouting ability tend to trade off seeding through persistence, while other species have greater reproductive performance and regenerate from seeds [37]. For the latter species, there are challenges that may arise when regenerating through seedling emergence because this may depend on a number of factors, such as seed viability, high seedling mortality during the dry season and climatic conditions [38]. Nonetheless, shoots produced by the cut stumps are undesirable because they have the ability to regrow into mature trees that may have competitive effects on the herbaceous layer [27]. To prevent tree stems from resprouting after tree cutting, the cut stems are frequently treated with chemical herbicides [39,40,41]. Cutting followed by an immediate application of herbicide to the stump can greatly reduce or prevent future sprouting in many woody and invasive species [41].

The competition for light and soil resources posed by high densities of woody plants may subsequently reduce the resprouting ability of cut stems [38]. Thus, gap formation through high intensities of tree removal may reduce the competition for resources of the remaining trees, which may consequently result in an increase in resprouting ability and vigour [38,42]. Resprouting vigour depends on the allocation of belowground stored reserves and the capacity to acquire new resources through photosynthesis [38,43], which may be enhanced via high intensities of tree removal. However, to our knowledge, the effects of different woody removal intensities on the resprouting patterns of woody species have not been studied before.

In general, the larger the stem, the greater the belowground resources that the plant has to support resprouting [44]. However, there is considerable variance in this relationship; some authors have found the opposite pattern [45], and some have found no relationship [46,47]. For instance, models developed by [48] to predict resprouting ability among oak trees in the central Appalachians in Pennsylvania (USA) show that white oak *Quercus alba* trees rapidly lost their resprouting abilities with increasing stem diameter. Meanwhile, ref. [49] suggested that the resprouting ability of woody plants as influenced by plant age is related to bud senescence. Additionally, thick bark in older trees may inhibit resprouting abilities through hindering bud emergence [32], particularly in systems that experience disturbances such as frequent fires [50,51]. Where faster growth allows trees to escape damage via frequent disturbances, resprouting ability may then decline with increasing size [52]. The tendency of young trees to be better resprouters than older trees is reported to be an effective adaptive strategy against frequent disturbances [31]. Therefore, these suggest that the effects of stem diameter on resprouting ability may be species-specific and may be related to the development of the root systems of species [50,53]. Consequently, differences in root systems among tree species may subsequently influence resprouting patterns and the efficacy of control methods.

Here, we examined the resprouting patterns of 12 dominant woody plant species at Roodeplaat Farm in the Gauteng Province of South Africa. We applied mechanical tree removal and herbicides to determine which of these two factors was most important for controlling woody plant encroachment. We sought to determine the combined effects of stem diameter, woody removal intensity (hereafter WRI) and herbicide application on the resprouting patterns of woody plants. To achieve these aims, we conducted a field experiment and made the following predictions: (1) herbicide application will result in reduced or no regrowth from cut stems, regardless of the species; (2) the resprouting ability will increase with increasing stem diameter because larger trees should have greater storage of belowground resources [36,45,54]; and (3) moderate and high WRIs will increase resprouting ability because of the substantial reduction in the competition for resources of the remaining trees.

## 2. Materials and Methods

### 2.1. Study Area

The study was conducted at the Roodeplaat Experimental Farm of the Agricultural Research Council (25°36′29″S, 28°2′08″ E) in Gauteng Province, South Africa. The farm is about 2100 ha, which is mostly used for livestock and game production. The vegetation type of the farm is the Marikana Thornveld [55]. The Marikana Thornveld is described as open *Vachellia karroo* (formerly *Acacia*) woodland occurring in valleys and slightly undulating plains and lowland hills [56]. *Vachellia karroo* and *Senegalia* (formerly *Acacia*) *caffra* [57], are among the major dominant woody plants on the farm. Other dominant woody plants include the *Euclea species*, *Vachellia* (formerly *Acacia) tortilis* and *Ziziphus mucronata*. The nomenclature of [56] for tree species was followed. The grass component of the site is characterised by *Digitaria eriantha*, *Eragrostis curvula*, *Heteropogon contortus*, *Melinis repens, Panicum maximum*, *Setaria sphacelata*, *Sporobolus africanus* and *Themeda triandra* [10]. We used the nomenclature of [58] for grass species. The study area is a mesic savanna with a mean annual rainfall of 687 mm, which largely falls between November and March. The minimum temperature during the winter season ranges between 2 and 16 °C, and the maximum summer temperature ranges between 20 and 29 °C. The experimental site was encroached at a density of 4065 ± 109 (mean ± SE) woody plants per ha^−1^. The experimental plots are located on sandy soils and have been permanently fenced to prohibit grazing since the establishment of the experiment in 2018.

### 2.2. Research Design

The study consisted of 20 plots of 30 m × 30 m each subjected to different intensities of tree removal. The tree densities were determined by doing a direct count of all trees in each plot. Trees were removed in October 2018 to the approximate equivalents of 10%, 20%, 50%, 75% and 100% (total clearing of the tree density) per plot, following [3]. The plots were close to each other and were separated by 5-m-wide fire breaks. Tree removal treatments were replicated four times and allocated randomly. The trees were cut with a chainsaw, and any accumulated sawdust or debris was removed from the cut stems. All trees were cut at a height of 0.25 m [25,27], and half the tree stems were treated with herbicide. The herbicide used contains Picloram as its active ingredient [39,59]. This herbicide is a water-soluble systemic herbicide with residual activity that acts through the roots and cut surfaces of woody plants [59]. The herbicide was applied at a minimum recommended concentration of 6 mL L^−1^ of water (Browser Herbicide^®^, Arysta Lifesciences, Tokyo, Japan) with crop oil added at 5 mL L^−1^. Tree stems were treated with herbicide within 15 min after felling during the growing season. A knapsack sprayer (Spraying Systems TG-1, Delavan CE 1) with a single solid-cone nozzle was used for herbicide application.

The combined effects of stem diameter, WRIs and herbicide application on the resprouting ability were examined on the following woody species that encroached on the study site: *Dichrostachys cinerea*, *Euclea crispa*, *Ehretia rigida*, *Gymnosporia buxifolia*, *Pappea capensis*, *Searsia lancea*, *Senegalia caffra*, *Vachellia karroo*, *V. nilotica*, *V. robusta*, *V. tortilis* and *Ziziphus mucronata*. To determine the regrowth patterns for each resprouting stem, the following variables were measured in each plot 9 months after tree felling towards the end of July 2019: (1) the total number of resprouting shoots per stem, (2) the shoot length of the leader shoot and (3) the shoot diameter of the leader shoot, measured at the base of the shoot. Shoot production was calculated as the number of shoots produced per stem diameter [25].

### 2.3. Data Analysis

Prior to analysis, data were log_10_-transformed to ensure a normal distribution of residuals, but the mean values and their associated standard errors were back-transformed after analysis. In addition, the data met all the MANOVA assumptions. We used multivariate analysis of covariance (MANCOVA) to test the effects of stem diameter, WRIs and herbicide application on the resprouting ability and vigour of the study plants. Shoot production, shoot length and shoot diameter were the dependent variables, with stem diameter as a covariate. MANCOVA was used to reduce the Type 1 error that may be caused by testing multiple dependent variables on the same subjects. We used Wilks’s λ test statistic to investigate the effect of treatments on resprouting parameters. When the MANCOVA was significant, we used univariate ANCOVA to identify factors that contributed to the significant MANCOVA, followed by a Bonferroni post hoc test among groups of each factor. We used linear regression to determine the relationship between the resprouting parameters of the trees and the stem diameter. The data were analysed separately for each species. IBM SPSS for Windows v. 26 [60] was used for all data analysis.

## 3. Results

There was no significant interaction among the resprouting parameters between the removal treatments and herbicide application for all species in this study (*p* > 0.05). We found no significant effect of the WRIs on the resprouting parameters for all species (*p* > 0.05). There was no significant effect of the covariate (stem diameter) on shoot length and diameter for all species (*p* > 0.05). However, there was a significant effect of stem diameter on shoot production for 10 of the 12 study species (*p* < 0.05).

**Figure 1:**
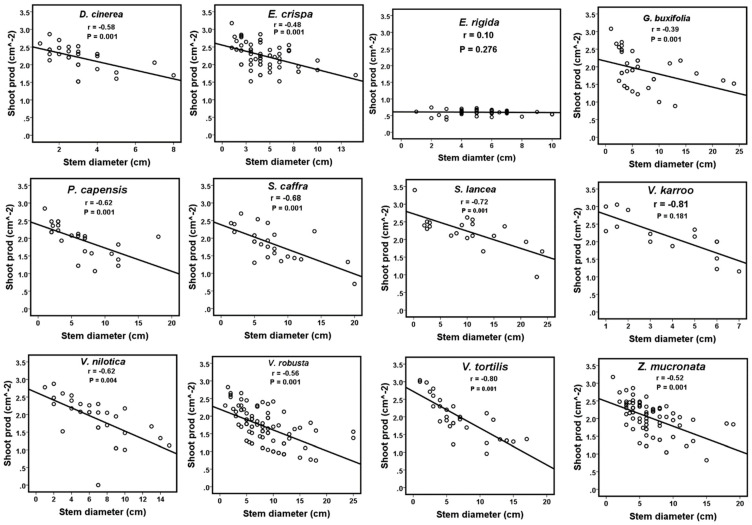
The relationship between stem diameter and new shoot production (“Shoot prod”) of the 12 study species: *D. cinerea, E. crispa*, *E. rigida*, *G. buxifolia*, *P. capensis*, *S. lancea*, *V. caffra*, *V. karroo*, *V. nilotica*, *V. robusta*, *V. tortilis* and *Z. mucronata*.

There was no significant effect of stem diameter on shoot production for *E. rigida* (*p* = 0.276) and *V. karroo* (*p* = 0.181) in the univariate ANCOVA. The results showed that *E. rigida* had the highest shoot production, while *V. robusta* had the lowest production of shoots (Table 1). Furthermore, we observed a significant negative relationship between stem diameter and shoot production for all the study species except for *E. rigida,* for which there was no clear pattern (Figure 1).

We found a significant effect of herbicide application on the resprouting patterns of four of the twelve study species (*p* < 0.05) (Table 2). Significant effects of herbicide application were found on *E. rigida*, *V. robusta*, *V. tortilis* and *Z. mucronata* (*p* < 0.05). A marginally significant effect (*p* < 0.058) of herbicide application was found for *D. cinerea* (Table 2).

## 4. Discussion

After bush clearing, tree regeneration is a major potential problem encountered in rangelands [1,25,61]. All the tree species in this study resprouted following cutting, demonstrating their ability to regenerate from damaged tissues. Our results are consistent with the results obtained in similar studies demonstrating woody plants’ abilities to resprout after disturbances [30,35,44,62]. The ability of woody plants to resprout after disturbances may be attributed to their stored resources [29,30,46]. The current study indicates that the trees examined in this study have the ability to regenerate after cutting, and thus, further stem treatment may be required to successfully control the plants to ensure a long-term reduction in woody populations [61].

We predicted that larger stems would show a stronger resprouting ability than smaller stems. However, we found that shoot production decreased with the increasing stem diameter of the study plants except for *E. rigida*. The findings of the current study are in line with [31,45,63], who demonstrated that the effectiveness of resprouting differs according to tree age, which is usually measured via stem diameter at the time of disturbance [31,45,63]. For example, several studies e.g., [45,48,62,64] have reported that tree species resprout as juveniles and lose their ability to resprout when they reach the adult stage. The causes of this resprouting pattern in woody species are unclear but are often assumed to arise from a combination of genetic, physiological and related anatomical changes that occur with the stage of tree development [29,48,64]. Moreover, the reduced resprouting ability of larger plants may be a consequence of the reduced production of non-structural carbohydrates [46]. Nonetheless, ref. [35] demonstrated that larger stems take longer to respond to the initial cutting but, once recovered, have the capacity to regrow at a rate faster than that of smaller stems. The study by [35] (39 months) lasted longer than our study (9 months), which may possibly explain why the results of his study and ours differed.

Herbicide application significantly reduced the resprouting abilities of *E. rigida*, *V. robusta* and *Z. mucronata*. Although herbicide application significantly reduced the shoot length of *V. tortilis*, it did not affect the resprouting ability (i.e., shoot production) or diameter of the leader shoot of this species. Furthermore, herbicide application had no significant effect on the resprouting ability of seven species that we tested (*E. crispa*, *G. buxifolia*, *P. capensis*, *S. lancea*, *V. caffra*, *V. karroo* and *V. nilotica*), which was inconsistent with our prediction that herbicide application would significantly reduce the resprouting ability of all cut stems, regardless of species. A possible reason for the inconsistency of the effects of herbicide application across species may be attributed to the equal concentration of Picloram applied to the cut stems and the time of application for each plant species. Elsewhere, ref. [65] found that the herbicide triclopyr amine applied at a 25% *v*/*v* (i.e., (volume of solute/volume of solution) × 100) concentration was not effective for the control of *Triadica sebifera*, an invasive woody species invading the southeastern United States. However, in the same study, ref. [65] found that the same amount of triclopyr amine was effective in controlling *Ligustrum sinense*, which invades the same area. Their results also showed that reducing the recommended concentrations of two herbicides (i.e., glyphosate and triclopyr amine) by 50% was effective for controlling *L. sinense*.

Reducing herbicide inputs into the environment is a desirable goal for land users globally [41] and particularly for resource-poor communal farmers. Thus, testing the amount of Picloram needed to kill certain woody species may be of importance for land users in southern African savannas. This will inform land managers of the optimal concentrations of Picloram to use on certain species. Moreover, the seasonal timing of herbicide application on cut stems has been reported to influence the subsequent resprouting of woody plants [41,66,67]. In our study, trees were cut and treated with herbicide during the wet season. However, ref. [41] demonstrated that woody plants are controlled better with herbicides during autumn (fall), when woody plants are not actively growing. Additionally, ref. [39] showed that using mixtures of several herbicides provided better control than using single herbicides because different herbicides have different physiological pathways and modes of action. Future studies should focus on testing the optimal concentrations and time (the wet or dry season) of applications of different herbicides needed to kill the tree stems of the species we examined.

Contrary to our expectations, moderate (50%) and high (75 and 100%) WRIs did not affect resprouting among the woody species. We attributed these findings to the distribution pattern of woody plants in savannas [18]. This is because savanna ecosystems are generally less dense compared to forest systems [52]. The competition for resources (particularly soil moisture) among savanna trees usually results in reduced plant densities and sizes and leads to a more regular pattern of tree distribution [68]. Thus, unlike in forest systems (e.g., [38]), high tree densities may not be an important determinant of resprouting success in savannas. However, more studies on the impact of tree cover on resprouting stems are needed, particularly in different savanna systems. Nonetheless, the findings of our study show that the resprouting ability of woody species is not dependent on tree densities.

## 5. Conclusions

The findings of this study provide evidence that woody species in this study area are capable of resprouting after disturbances. Herbicide application did not significantly reduce the resprouting ability of all the study plants. These results suggest that higher concentrations of herbicides, particularly Picloram, may be required to successfully prevent cut stems from resprouting in other tree species. This may, however, pose a challenge for resource-constrained farmers who seldom have access to sufficient funds to finance the control of woody plants. We warrant research that will test different concentrations of Picloram and the timing of application required for successful reduction in the resprouting ability of woody species (i.e., *D. cinerea*, *E. crispa*, *G. buxifolia*, *P. capensis*, *S. lancea*, *V. caffra*, *V. karroo* and *V. nilotica*) that were not significantly affected by herbicide application. Nonetheless, the resprouting ability reduced with an increasing stump diameter. Consequently, woody plants are more likely to resprout and survive disturbances as juveniles than as adults. This suggests the rejection of the prediction that the resprouting ability increases with an increasing stem diameter. In addition, a moderate to high density of tree removal did not increase the resprouting ability, thus indicating that the tree canopy cover is not an important determinant of resprouting success in savannas.

## Figures and Tables

**Table 1:** Mean (± S.E.) stem diameter, number of stems recorded (n) and overall shoot production.

Species	Stem Diameter (cm)	n	Shoot Production (cm^−2^)
*D. cinerea*	3.8 ± 0.2	72	81.1 ± 16.4
*E. crispa*	4.7 ± 0.2	151	102.4 ± 15.9
*E. rigida*	5.2 ± 0.1	100	157.4 ± 20.7
*G. buxifolia*	6.7 ± 0.6	82	65.3 ± 18.3
*P. capensis*	6.9 ± 0.7	35	101.8 ± 23.8
*S. lancea*	9.7 ± 0.8	42	147.1 ± 60.4
*S. caffra*	7.8 ± 0.7	40	70.5 ± 17.4
*V. karroo*	4.8 ± 0.3	55	79.4 ± 30.8
*V. nilotica*	8.4 ± 0.5	70	62.1 ± 15.4
*V. robusta*	9.6 ± 0.3	201	41.4 ± 6.4
*V. tortilis*	9.1 ± 0.8	47	144.8 ± 39.1
*Z. mucronata*	7.1 ± 0.3	140	93.1 ± 14.9

**Table 2:** The effect of herbicide application on the means of each of the following: number of leaves, shoot diameter (cm), length of the leader shoot (longest shoot) (cm) and shoot production of 12 tree species. Significant differences in the ANOVA results are denoted with an *. The species names are *Dichrostachys cinerea*, *Euclea crispa*, *Ehretia rigida*, *Gymnosporia buxifolia*, *Pappea capensis*, *Searsia lancea*, *Senegalia caffra*, *Vachellia karroo*, *V. nilotica*, *V. robusta*, *V. tortilis* and *Ziziphus mucronata*.

Species	Treatment	Significance of Wilks’s λ in MANCOVA (*p*-Value)	Diameter of the Leader Shoot (Mean ± SE)	Length of the Leader Shoot (Mean ± SE)	Shoot Production (Mean ± SE)
*D. cinerea*	Herbicide Control	0.058	0.03 ± 0.01 0.49 ± 0.07	6.20 ± 3.13 68.03 ± 9.39	13.5 ± 0.8 193.3 ± 32.6 *
*E. crispa*	Herbicide Control	0.225	0.03 ± 0.01 0.31 ± 0.04	3.79 ± 1.44 37.60 ± 3.61	25.32 ± 10.67 204.44 ± 29.79
*E. rigida*	Herbicide Control	0.001	0.04 ± 701.24 0.75 ± 0.07 *	4.81 ± 3.22 65.85 ± 5.93 *	20.6 ± 9.2 317.9 ± 29.6 *
*G. buxifolia*	Herbicide Control	0.138	0.05 ± 0.02 0.33 ± 0.05	2.35 ± 1.03 22.76 ± 3.34	9.0 ± 3.7 149.8 ± 42.4
*P. capensis*	Herbicide Control	0.099	0.08 ± 0.02 0.37 ± 0.09	6.54 ± 2.58 119.66 ± 87.26	44.5 ± 21.8 156.2 ± 37.8
*S. lancea*	Herbicide Control	0.347	0.12 ± 0.06 2.81 ± 1.82	9.64 ± 4.89 89.00 ± 9.71	114.7 ± 100.1 194.9 ± 28.5
*V. caffra*	Herbicide Control	0.122	0.21 ± 0.07 0.45 ± 0.08	3.70 ± 10.06 62.29 ± 10.19	28.1 ± 9.7 134.3 ± 36.9
*V. karroo*	Herbicide Control	0.158	0.01 ± 0.01 0.35 ± 0.07	1.89 ± 1.36 42.22 ± 4.68	10.1 ± 6.2 221.7 ± 85.3
*V. nilotica*	Herbicide Control	0.083	0.04 ± 0.03 0.36 ± 0.05	2.09 ± 1.23 42.28 ± 5.45	3.9 ± 2.9 130.1 ± 29.5
*V. robusta*	Herbicide Control	0.004	0.09 ± 0.02 0.42 ± 0.04 *	5.84 ± 1.15 39.64 ± 3.08 *	6.8 ± 1.7 155.7 ± 15.2 *
*V. tortilis*	Herbicide Control	0.038	0.15 ± 0.05 0.65 ± 0.12	17.67 ± 4.75 61.94 ± 6.67 *	98.6 ± 48.2 212.9 ± 63.5
*Z. mucronata*	Herbicide Control	0.001	0.07 ± 0.02 1.05 ± 0.08 *	7.02 ± 2.44 104.92 ± 5.89 *	4.3 ± 1.5 192.4 ± 26.8 *

## Data Availability

The data that support this study will be shared upon reasonable request to the corresponding author.
